# A photon counting and a squeezing measurement method by the exact absorption and dispersion spectrum of Λ-type Atoms

**DOI:** 10.1186/s40064-016-3014-7

**Published:** 2016-08-24

**Authors:** Ghasem Naeimi, Samira Alipour, Siamak Khademi

**Affiliations:** 1 Physics Groups, Qazvin Branch, Islamic Azad University, Qazvin, Iran; 2 Department of Physics, University of Zanjan, 6th Km of Tabriz Road, Zanjan, P.O. Box 38791-45371, Iran

**Keywords:** Electromagnetically induced transparency, Exact method, Non-demolition, Photon counting method, Measurement of the squeezing parameter

## Abstract

Recently, the master equations for the interaction of two-mode photons with a three-level Λ-type atom are exactly solved for the coherence terms. In this paper the exact absorption spectrum is applied for the presentation of a non-demolition photon counting method, for a few number of coupling photons, and its benefits are discussed. The exact scheme is also applied where the coupling photons are squeezed and the photon counting method is also developed for the measurement of the squeezing parameter of the coupling photons.

## Background

Electromagnetically induced transparency (EIT) has been theoretically introduced by Kocharovskaya and Khanin (1988) and experimentally observed, by Harris et al. (1990) and Harris (1997). Recently, many authors have been interested in studying EIT and its applications (Sargsyan et al. 2012; Hong-Wei and Xian-Wu 2012; Marangos 1998; Deng and Payne 2005; Chenguang and Zhang 2008; Jafari et al. 2011; Sahrai et al. 2011; Rabiei et al. 2011; Sahrai et al. 2010a, b). EIT is widely studied for different systems, e.g. V, Λ and cascade three-level atoms (Olson and Mayer 2009; Fleischhauer et al. 2005; Lazoudis et al. 2010, 2011) and many other atoms with more levels (Bai et al. 2013; Joshi and Xiao 2003). Many alkali atoms, e.g., Rydberg Rubidium atom, have been also experimentally used (Petrosyan et al. 2011; Wang et al. 2004). Properties of the electromagnetic fields interacting with a three-level Λ-type atom were studied in the semi-classical (Kocharovskaya and Khanin 1988; Harris et al. 1990; Harris 1997; Scully and Zubairy 1997; Dantan et al. 2012) and full-quantum (Wang et al. 1992; Akamatsu et al. 2004; Johnsson and Fleischhauer 2002) models by a weak field approximation (WFA) method. In WFA the possibility of research on the small intensity of coupling field disappears because the coupling field should have larger intensity compare to the probe field to establish approximation. The authors of this paper presented an exact analytical solution for multilevel systems that interact with the probe and quantized coupling fields (which is also applied for small intensity of coupling field) (Khademi et al. 2015). The EIT with the quantized fields in opto-cavity mechanics is another example for the full-quantum approach which is studied by Huang and Agarwal (2011). The destructive detection of photons has been investigated theoretically and experimentally. But non-demolition detection of photons (Braginsky and Khalili 1996) has until now been an interesting ultimate goal of some optical measurement methods (Grangier et al. 1998). In 2012, Serge Haroche and coworkers have been shown (Sayrin et al. 2012) that interaction of microwave photons, trapped in a superconducting cavity, with Rydberg atoms crossing the cavity, illustrates a non-demolition photon counting. In 2013, Andreas Raiser (Raiser et al. 2013) presents another method for non-demolition detection of photons which are passing through a superconductive cavity resonator that includes rubidium atoms. Haroche et al. (Sayrin et al. 2012) and Naeimi et al. (2013) investigated a photon counting and squeezing parameter measurement (for photons trapped in a quantum cavity) by measure the properties of a beam of atoms interacted with an array of cavities. But photon counting by measure the properties of another photons (or field) which are passing through the cavity, have never been investigated to our best of knowledge. In this paper, we present an exact analytical non-demolition photon counting method (for photons inside a cavity) by investigating the absorption profile of probe field. A full-quantum model of EIT is investigated for an ensemble of Λ-type three-level atoms, in which the probe and coupling fields are quantize. Interaction of a Λ-type three-level atom with the quantized electromagnetic fields is investigated using the Jaynes–Cummings model (Khademi et al. 2015). The Jaynes–Cummings interaction Hamiltonian is applied for each of the coupled levels. In this case, the exact master equations are investigated and solved in a steady-state without any WFA (Khademi et al. 2015). An exact form of absorption and dispersion spectra are obtained for the probe fields which are not generally weaker than the coupling field. It is shown that the EIT obtained for the probe fields is either weaker or stronger than that of the coupling field.

Moreover, profile of the absorption and dispersion spectra are shown to depend on the number of coupling photons so that the number of coupling photons could be measured using the absorption spectrum of the probe photons. This scheme is applied for the presentation of a non-demolition photon counting method. The present method is applied to the squeezed coupling photons. Straightforwardly, it is shown that the exact absorption and dispersion spectra drastically depended on squeezing parameter of the coupling photons. This scheme is also applied for presenting measurement of the squeezing parameter.

In “A review on the exact model” section, a review on the exact model of the full-quantum interaction of quantized electromagnetic fields with a Λ-type three-level atom will be presented. More details are found in reference (Khademi et al. 2015). The master equations in the steady-state, their exact solutions, a schematic experimental setup and notations are also introduced. “Photon counting by an ensemble of Λ-type three-level atom” section is devoted to a photon counting method in terms of the measurement of absorption spectrum. In “Measuring squeezing of trapped coupling photons” section, the exact probe coherence term is obtained where the coupling photons are squeezed. It is shown that the squeezing parameter is also measurable by the measurement of absorption and dispersion spectrum. The last section is devoted to the “Conclusions”.

## A review on the exact model

In this section a review on the exact model of a three-level Λ-type atom interacting with two quantized electromagnetic field (Khademi et al. 2015) is presented. The master equations, notations, experimental setup and some solutions and results are used in the next sections.

**Fig. 1:**
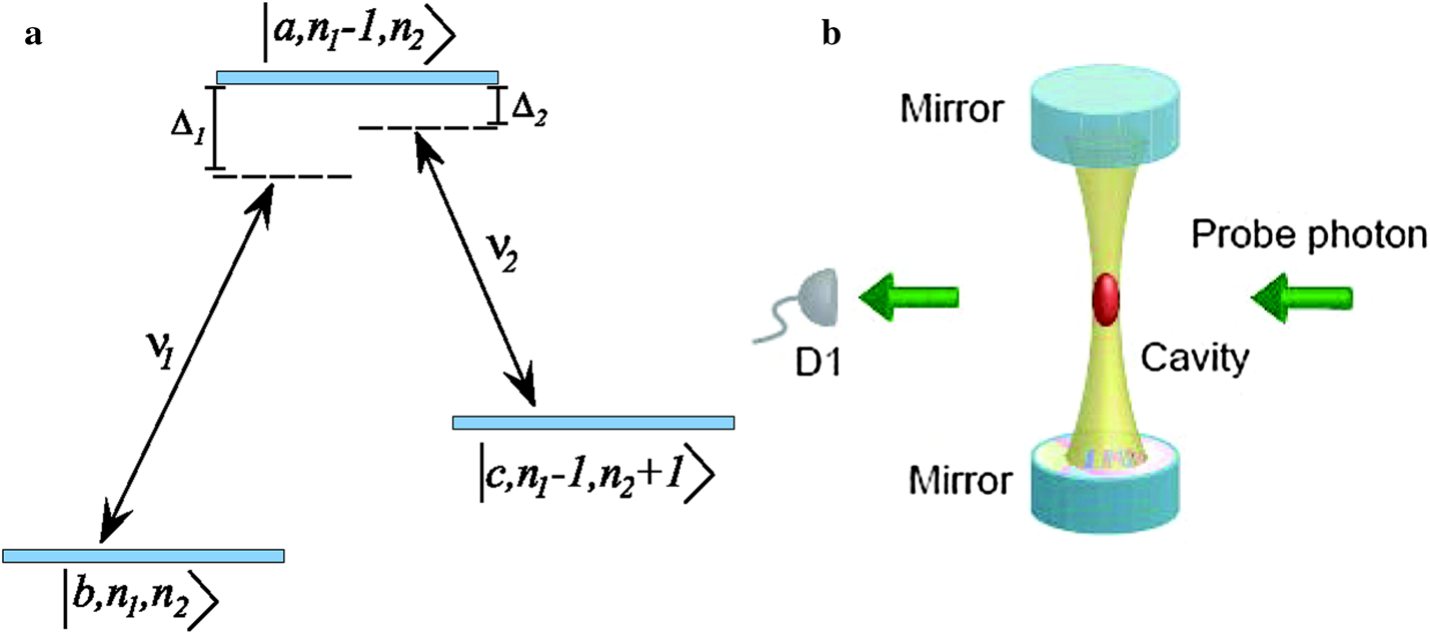
(Color online) (**a**) A Λ-type three-level atom interacting with two electromagnetic fields with frequencies $\nu_{1}$ and $\nu_{2}$. The *red spot* is an ensemble of atoms trapped and strongly coupled with the quantum cavity. The quantized probe photons are passed through the cavity and counted by D1 after interaction with the ensemble of atoms (Khademi et al. 2015)

Suppose that, in cavity quantum electrodynamics, the quantized probe and coupling fields (photons) interact with a three-level Λ-type atom (see Fig. 1a). The interaction Hamiltonian of this system in the interaction picture is given by:

1 $${\text{V}} = - \hbar {\text{g}}_{ 1} \left[ {\sigma_{\text{ab}} {\text{a}}_{ 1} {\text{e}}^{{{\text{i}}\Delta_{ 1} {\text{t}}}} + {\text{a}}_{ 1}^{\dag } \sigma_{\text{ba}} {\text{e}}^{{ - {\text{i}}\Delta_{ 1} {\text{t}}}} } \right] - \hbar {\text{g}}_{ 2} \left[ {\sigma_{\text{ac}} {\text{a}}_{ 2} {\text{e}}^{{{\text{i}}\Delta_{ 2} {\text{t}}}} +{\text{ a}}_{ 2}^{\dag } \sigma_{\text{ca}} {\text{e}}^{{ - {\text{i}}\Delta_{ 2} {\text{t}}}} } \right],$$

where $g_{1} = \wp_{ab} \cdot \,\hat{\varepsilon }_{1} E_{1} /\hbar$ and $g_{2} = \wp_{ac} \,\hat{\varepsilon }_{2} E_{2} /\hbar$ are interaction strength of the probe and coupling fields, respectively, and $E_{i} = (\hbar \nu_{i} /2\varepsilon_{0} {\text{v}})^{1/2}$. In this case, ${\text{v}}$ is cavity volume and $\wp_{ab} = {\text{e}}\left\langle {\text{a}} \right|{\text{r}}\left| {\text{b}} \right\rangle$ and $\wp_{ac} = {\text{e}}\left\langle {\text{a}} \right|{\text{r}}\left| {\text{c}} \right\rangle$ are matrix elements of atomic dipole moments, induced by the electromagnetic fields. $\hat{a}_{1} \left( {\hat{a}_{1}^{{{\dag }}} } \right)$ and $\hat{a}_{2} \left( {\hat{a}_{2}^{{{\dag }}} } \right)$ are annihilation (creation) operators for the probe and coupling photons, respectively. $\sigma_{ij} = \left| i \right\rangle \left\langle j \right|$ is atomic transition operator from $\left| j \right\rangle \to \left| i \right\rangle$. In Eq. (1), $\Delta_{1} = \omega_{ab} - \nu_{1} (\Delta_{2} = \omega_{ac} - \nu_{2} )$ is detuning between the frequency of probe (coupling) and the atomic transition frequency $\left| a \right\rangle \to \left| b \right\rangle \, (\left| a \right\rangle \to \left| c \right\rangle )$.

Assume the system is initially in the ground state $\left| b \right\rangle$ and the electromagnetic fields for the probe and coupling fields are in the states $\left| {n_{1} } \right\rangle$ and $\left| {n_{2} } \right\rangle$, respectively. Therefore, the initial state of total system is given by $\left| {b,n_{1} ,n_{2} } \right\rangle$. After an atom–field interaction, one photon with frequency of $\nu_{1}$ is absorbed and the atom is then transited into the higher level $\left| a \right\rangle$ and state of the total system changes to $\left| {a,n_{1} - 1,n_{2} } \right\rangle$. Due to the spontaneous or induced emission, the atom in the state $\left| a \right\rangle$ is transited into another level $\left| c \right\rangle$ and one photon with frequency of $\nu_{2}$ is emitted and state of the total system changes to $\left| {c,n_{1} - 1,n_{2} + 1} \right\rangle$. The master equations are obtained as:

2 $$\dot{\tilde{\rho }}_{aa} = - \left( {\gamma_{1} + \gamma_{2} } \right)\tilde{\rho }_{aa} + ig_{1} \sqrt {n_{1} } \left( {\tilde{\rho }_{ba} - \tilde{\rho }_{ab} } \right) + ig_{2} \sqrt {n_{2} + 1} \left( {\tilde{\rho }_{ca} - \tilde{\rho }_{ac} } \right)$$

3 $$\dot{\tilde{\rho }}_{bb} = \gamma_{1} \tilde{\rho }_{aa} + \gamma_{3} \tilde{\rho }_{cc} + ig_{1} \sqrt {n_{1} } \left( {\tilde{\rho }_{ab} - \tilde{\rho }_{ba} } \right)$$

4 $$\dot{\tilde{\rho }}_{cc} = \gamma_{2} \tilde{\rho }_{aa} - \gamma_{3} \tilde{\rho }_{cc} + ig_{2} \sqrt {n_{2} + 1} \left( {\tilde{\rho }_{ac} - \tilde{\rho }_{ca} } \right)$$

5 $$\dot{\tilde{\rho }}_{ab} = - \tfrac{1}{2}\left( {\gamma_{1} + 2i\Delta_{1} } \right)\tilde{\rho }_{ab} + ig_{1} \sqrt {n_{1} } \left( {\tilde{\rho }_{bb} - \tilde{\rho }_{aa} } \right) + ig_{2} \sqrt {n_{2} + 1} \tilde{\rho }_{cb}$$

6 $$\dot{\tilde{\rho }}_{ac} = - \tfrac{1}{2}\left( {\gamma_{2} + 2i\Delta_{2} } \right)\tilde{\rho }_{ac} + ig_{1} \sqrt {n_{1} } \tilde{\rho }_{bc} + ig_{2} \sqrt {n_{2} + 1} \left( {\tilde{\rho }_{cc} - \tilde{\rho }_{aa} } \right)$$

7 $$\dot{\tilde{\rho }}_{bc} = - \tfrac{1}{2}\left( {\gamma_{3} + - 2i\left( {\Delta_{2} - \Delta_{1} } \right)} \right)\tilde{\rho }_{bc} + ig_{1} \sqrt {n_{1} } \tilde{\rho }_{ac} - ig_{2} \sqrt {n_{2} + 1} \tilde{\rho }_{ba}$$

where $\rho_{ij} = \rho_{ji}^{*}$, $\gamma_{1} = \varGamma_{ab} ,\gamma_{2} = \varGamma_{ac}$ and $\gamma_{3} = \varGamma_{cb}$ are spontaneous decay rates. To obtain Eqs. (2)–(7), the rotating frame transformations: $\tilde{\rho }_{ab} = \rho_{ab} \exp \{ - i\Delta_{1} t\}$, $\tilde{\rho }_{ac} = \rho_{ac} \exp \{ - i\Delta_{2} t\}$ and $\tilde{\rho }_{cb} = \rho_{cb} \exp \{ i(\Delta {}_{2} - \Delta_{1} )t\}$ are applied.

**Fig. 2:**
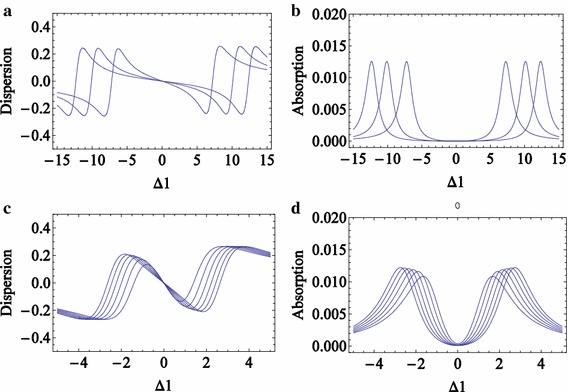
(Color online) **a**, **b** are the dispersion and absorption of the probe field in terms of its detuning for a large number of coupling photons ($n_{2} = 50,100,150$); **c**, **d** are the dispersion and absorption of the probe field for a small number of coupling photons ($n_{2} = 0,1,2,3,4,5$). They are plotted versus detuning of probe field $\Delta_{1}$ where other parameters are $\gamma_{1} = 0.1,\gamma_{2} = 0.1,\gamma_{3} = 0.001,n_{1} = 1,\Delta_{2} = 0,g_{1} = g_{2} = 1$

An ensemble of cold three-level atoms is prepared by an optical pumping initially in the state $\left| b \right\rangle$. The quantum cavity is filled with the three-level cold atoms as well as the $n_{2}$ number of coupling photons. The coupling photons are strongly coupled with the quantum cavity electrodynamics. The probe photons are individually injected into the cavity and absorptive atoms. Absorption of the probe photons is controlled by the number of coupling photons $n_{2}$ and measured by the detector D1. This experiment would be frequently performed for a specific number of the coupling photons trapped in the cavity. Absorption spectrums for different numbers of coupling photons are plotted in Fig. 2b, d.

The master Eqs. (2)–(7) are exactly solved in the steady-state to obtain the exact coherence term $\tilde{\rho }_{ab}$ (Khademi et al. 2015). The result is arranged in terms of different orders of the probe detuning in the numerator and denominator of exact $\tilde{\rho }_{ab}$. The compact result could be written as:

8 $$\tilde{\rho }_{ab} = \frac{{2g_{1} \sqrt {n_{1} } \left( {iZ_{0} + Z_{1} \Delta_{1} + iZ_{2} \Delta_{1}^{2} + Z_{3} \Delta_{1}^{3} } \right)}}{{K_{0} + K_{2} \Delta_{1}^{2} + K_{4} \Delta_{1}^{4} }},$$

where

9 $$ \begin{aligned} Z_{0} & = \gamma_{3} \left( {4g_{1}^{2} n_{1} \gamma_{1} + 4g_{2}^{2} \gamma_{2} \left( {n_{2} + 1} \right) + \gamma_{1} \gamma_{2} \gamma_{3} } \right)\bigg(4g_{2}^{2} (\gamma_{1} + \gamma_{3} ) \\ & \quad \times \left( {n_{2} + 1} \right) + \left( {\gamma_{1} + \gamma_{2} } \right)\left( {4g_{1}^{2} n_{1} + \gamma_{2} \gamma_{3} } \right)\bigg), \\ \end{aligned} $$

10 $$\begin{aligned} Z_{1} & = \left( { - 32g_{2}^{4} \left( {n_{2} + 1} \right)^{2} \gamma_{2} \left( {\gamma_{1} + \gamma_{3} } \right) + 2\gamma_{3} \left( {\gamma_{1} + \gamma_{2} } \right)\left( {4g_{1}^{2} n_{1} + \gamma_{1} \gamma_{3} } \right)^{2} } \right. \\ & \quad - 8g_{2}^{2} \left( {n_{2} + 1} \right)\left( {\gamma_{2} \left( {\gamma_{2} + \gamma_{3} } \right)\gamma_{3} \left( {\gamma_{1} + \gamma_{2} + \gamma_{3} } \right)} \right. \\ & \quad - 4g_{1}^{2} n_{1} \left. {\left. {\left( { - \gamma_{2}^{2} + \gamma_{3} \left( {\gamma_{1} + \gamma_{2} } \right) + \gamma_{3}^{2} } \right)} \right)} \right), \\ \end{aligned}$$

11 $$Z_{2} = 4\gamma_{1} \gamma_{2} (\gamma_{2} \gamma_{3} (\gamma_{1} + \gamma_{2} ) + 4g_{2}^{2} (n_{2} + 1)(\gamma_{1} + \gamma_{3} )),$$

12 $$Z_{3} = 8\gamma_{2} \left( { - \gamma_{2} \gamma_{3} \left( {\gamma_{1} + \gamma_{2} } \right) - 4g_{2}^{2} \left( {n_{2} + 1} \right)\left( {\gamma_{1} + \gamma_{3} } \right)} \right),$$

13 $$\begin{aligned} K_{0} & = \left( {4g_{1}^{2} n_{1} \gamma_{1} + 4g_{2}^{2} \gamma_{2} \left( {n_{2} + 1} \right) + \gamma_{1} \gamma_{2} \gamma_{3} } \right)\left( {16g_{2}^{4} \left( {n_{2} + 1} \right)^{2} \left( {\gamma_{1} + \gamma_{3} } \right)} \right. \\ & \quad + \left( {4g_{1}^{2} n_{1} + \gamma_{2} \gamma_{3} } \right)\left( {\gamma_{1} \gamma_{3} \left( {\gamma_{1} + \gamma_{2} } \right) + 4g_{1}^{2} n_{1} \left( {\gamma_{2} + 2\gamma_{3} } \right)} \right) \\ & \quad \left. { + 4g_{2}^{2} \left( {n_{2} + 1} \right)\left( {4g_{1}^{2} n_{1} \left( {\gamma_{1} + \gamma_{2} } \right) + \gamma_{3} \left( {\gamma_{1}^{2} + \gamma_{2}^{2} + \gamma_{1} \left( {\gamma_{2} + \gamma_{3} } \right)} \right)} \right)} \right) \\ \end{aligned}$$

14 $$\begin{aligned} K_{2} & = 4\left( {16g_{1}^{4} n_{1}^{2} \gamma_{3} \left( {\gamma_{1} + \gamma_{2} } \right) - 32g_{2}^{4} \left( {n_{2} + 1} \right)^{2} \gamma_{2} \left( {\gamma_{1} + \gamma_{3} } \right)} \right. \\ & \quad + \gamma_{2}^{2} \left( {\gamma_{1} + \gamma_{2} } \right)\gamma_{3} \left( {\gamma_{1}^{2} + \gamma_{3}^{2} } \right) + 4g_{1}^{2} n_{1} \gamma_{2} \left( {\gamma_{2}^{2} \gamma_{1} + 2\gamma_{1} \gamma_{2} \gamma_{3} } \right. \\ & \quad + 2\left( {\gamma_{1} + \gamma_{2} \gamma_{3}^{2} } \right) + 4g_{2}^{2} \left( {n_{2} + 1} \right)\left( {\gamma_{2} \left( {\gamma_{1}^{3} + \gamma_{1}^{2} \gamma_{3} - 2\gamma_{2}^{2} \gamma_{3} + \gamma_{3}^{3} } \right)} \right. \\ & \quad + \gamma_{1} \gamma_{3} \left. {\left( { - 2\gamma_{2} + \gamma_{3} } \right)} \right) + 4g_{1}^{2} n_{1} \left( {\gamma_{2}^{2} + 3\gamma_{2} \gamma_{3} + \gamma_{3}^{2} } \right. \\ & \quad \left. {\left. {\left. { + \gamma_{1} \left( {2\gamma_{2} + \gamma_{3} } \right)} \right)} \right)} \right), \\ \end{aligned}$$

15 $$K_{4} = 16\gamma_{2} \left( {\gamma_{2} \gamma_{3} \left( {\gamma_{1} + \gamma_{2} } \right) + 4g_{2}^{2} \left( {n_{2} + 1} \right)\left( {\gamma_{1} + \gamma_{3} } \right)} \right),$$

are the real parameters (Khademi et al. 2015). Dispersion and absorption of the coherence term (8) are proportional to

16 $$\text{Re} [\tilde{\rho }_{ab} ] = \frac{{2g_{1} \sqrt {n_{1} } (Z_{1} \Delta_{1} + Z_{3} \Delta_{1}^{3} )}}{{K_{0} + K_{2} \Delta_{1}^{2} + K_{4} \Delta_{1}^{4} }},$$

17 $$\text{Im} [\tilde{\rho }_{ab} ] = \frac{{2g_{1} \sqrt {n_{1} } (Z_{0} \Delta_{1} + Z_{2} \Delta_{1}^{2} )}}{{K_{0} + K_{2} \Delta_{1}^{2} + K_{4} \Delta_{1}^{4} }},$$

respectively.

The real and imaginary parts of $\tilde{\rho }_{ab}$ are plotted in Fig. 2a–d for large and small numbers of coupling photons. It is shown that the detuning of the absorption peaks (DAPs) $\Delta_{1}$, increases with increasing the number of coupling photons.

In the next section, a photon counting method based on the exact form of absorption spectrum of the probe field, which is derived from Eq. (17), is presented.

## Photon counting by an ensemble of Λ-type three-level atom

In this section, a non-demolition photon counting method is presented for measuring the number of coupling photons which are trapped in a quantum cavity and interact with an ensemble of three-level **Λ**-type atoms.

It is worthwhile to estimate the number of probe photons which is required to determine the probe absorption peak. As an example, the cavity field decay rate can be estimated as $\kappa = 5\pi \;{\text{MHz}}$ (Raiser et al. 2013) and the coupling photons inside a high Q-factor cavity will not decay as soon as 0.1 μs. A traveling time for a probe photon passing through a cavity with dimensions about a few millimeters is also about 3 ps. Approximately, $2 \times 10^{4}$ of probe photons are passing through the cavity meanwhile the coupling photons are trapped. This number of photons is sufficient to have a good precision to determine the absorption and dispersion curves in different detuning.

**Fig. 3:**
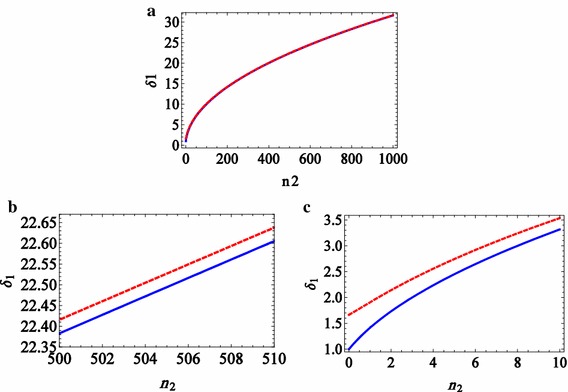
(Color online) The DAPs versus the number of coupling photons for WFA (*solid blue line*) and exact (*dashed red line*) methods; **a** for the large number of coupling photons, the WFA and exact methods are very similar, **b** for the large number of coupling photons, WFA and exact methods have a fine different result, **c** for the small number of coupling photons, WFA and exact methods have different results

It is clear in Eqs. (8)–(17) and Fig. 2 that the profile and the probe DAP in the absorption spectrum depend on the number of coupling photons. The derivative of the imaginary part of the coherence term, Eq. (17), with respect to $\Delta_{1}$ can be set to zero:

18 $$\frac{d}{{d\Delta_{1} }}\text{Im} [\tilde{\rho }_{ab} ] = 0,$$

to obtain the DAP $\delta_{1} (n_{2} ) = \Delta_{1Max.Abs.} .$ DAPs are nonlinearly increased by the number of coupling photons $n_{2}$, as plotted in Fig. 3a–c for large and small numbers of coupling photons. Figure 3a presents the relation between measurable DAPs and the large number of coupling photons.

Although Fig. 3a shows the same behaviour in the exact and WFA methods for a large number of coupling photons, there is a fine difference which is shown in Fig. 3b. But, Fig. 3c shows the difference between WFA and exact methods for the small number of coupling photons. In this condition, the exact method has more benefits than the WFA methods.

A considerable difference between the plots in Fig. 3b, c indicates that the exact method in the full-quantum model provides more correct photon numbers, even for a few number of coupling photons. Furthermore, measurement of absorption spectrum versus detuning of the probe field is a simpler method compared with other photon counting schemes. It is also a non-demolition method for weak probe fields.

## Measuring squeezing of trapped coupling photons

Another application of the full-quantum interaction of two-mode photons with three-level Λ-type atoms is in measurement of squeezing parameter of coupling photons. Supposed the trapped coupling photons are squeezed $\left| {n_{2} ,\xi } \right\rangle = \hat{S}(\xi )\left| {n_{2} } \right\rangle$ with a squeezing operator $\hat{S}(\xi ) = \exp (\tfrac{1}{2}\xi^{ * } \hat{a}^{2} - \tfrac{1}{2}\xi \hat{a}^{\dag 2} )$, where $\xi = r\exp \{ i\beta \} .$*r* and $\beta$ are also the squeezing parameter and squeezing phase, respectively.

In this case, the interaction Hamiltonian in the interaction picture is given by (“Appendix”)

19 $${\text{V}} = - \hbar {\text{g}}_{1} \left[ {\sigma_{\text{ab}} {\text{a}}_{1} {\text{e}}^{{{\text{i}}\Delta_{1} {\text{t}}}} + {\text{a}}_{1} {\kern 1pt}^{{{\dag }}} \sigma_{\text{ba}} {\text{e}}^{{ - {\text{i}}\Delta_{1} {\text{t}}}} } \right] - \hbar {\text{g}}_{2} \cosh ({\text{r}})\left[ {\sigma_{\text{ac}} {\text{a}}_{2} {\text{e}}^{{{\text{i}}\Delta_{2} {\text{t}}}} + {\text{a}}_{2} {\kern 1pt}^{{{\dag }}} \sigma_{\text{ca}} {\text{e}}^{{ - {\text{i}}\Delta_{2} {\text{t}}}} } \right],$$

Equation (19) is similar to Eq. (1) where ${\text{g}}_{ 2} \to {\text{g}}_{ 2} {\text{cosh(r)}} .$ Substituting ${\text{g}}_{ 2} {\text{cosh(r)}}$ instead of ${\text{g}}_{ 2}$ in Eqs. (2)–(7) and, after some calculations, the exact probe coherence term is given in terms of squeezing parameter $r$ as:

20 $$\tilde{\rho }_{ab} = \frac{{2g_{1} \sqrt {n_{1} } \left( {iL_{0} + L_{2} g_{2}^{2} \cosh^{2} \left( r \right) + L_{4} g_{2}^{4} \cosh^{4} \left( r \right)} \right)}}{{M_{0} + M_{2} g_{2}^{2} \cosh^{2} \left( r \right) + M_{4} g_{2}^{2} \cosh^{4} \left( r \right) + M_{6} g_{2}^{6} \cosh^{6} \left( r \right)}},$$

where

21 $$L_{0} = \gamma_{3} \left( {\gamma_{1} + \gamma_{2} } \right)\left( {i\gamma_{1} + 2\Delta_{1} } \right)\left( {\left( {4g_{1}^{2} n_{1} + \gamma_{1} \gamma_{3} } \right)^{2} + 4\gamma_{2}^{2} \Delta_{1}^{2} } \right),$$

22 $$\begin{aligned} L_{2} & = 4\left( {n_{2} + 1} \right)\left( {i\gamma_{3} \left( {4g_{2}^{2} n_{1} + \gamma_{2} \gamma_{3} } \right)\left( {\gamma_{1}^{2} + \gamma_{2}^{2} + \gamma_{1} \left( {\gamma_{2} + \gamma_{3} } \right)} \right)} \right. \\ & \quad + 2\left( { - \gamma_{2} \gamma_{3} \left( {\gamma_{2} - \gamma_{3} } \right)\left( {\gamma_{1} + \gamma_{2} + \gamma_{3} } \right) - 4g_{1}^{2} n_{1} } \right)\left( { - \gamma_{2}^{2} + \gamma_{2} \gamma_{3} } \right. \\ & \quad \left.\left.+ \gamma_{3} \left( {\gamma_{1} + \gamma_{3} } \right)\right)\right)\Delta_{1} + 4i\gamma_{3} \gamma_{1} \left( {\gamma_{1} + \gamma_{3} } \right)\Delta_{1}^{2} + 8\gamma_{2} \left( {\gamma_{1} + \gamma_{3} } \right)\left. {\Delta_{1}^{3} } \right), \\ \end{aligned}$$

23 $$L_{4} = 16i\left( {n_{2} + 1} \right)^{2} \gamma_{2} \left( {\gamma_{1} + \gamma_{3} } \right) + \left( {2i\Delta_{1} + \gamma_{3} } \right),$$

24 $$\begin{aligned} M_{0} & = \left( {\left( {4g_{1}^{2} n_{1} + \gamma_{2} \gamma_{3} } \right)^{2} + 4\gamma_{2}^{2} \Delta_{1}^{2} } \right)\left( {4g_{1}^{2} n_{1} \gamma_{1} \left( {\gamma_{2} + 2\gamma_{3} } \right)} \right. \\ & \quad + \gamma_{3} \left.\left( {\gamma_{1} + \gamma_{2} } \right)\left( {\gamma_{1}^{2} + 4\Delta_{1}^{2} } \right)\right), \end{aligned}$$

25 $$\begin{aligned} M_{2} & = 4\left( {n_{2} + 1} \right)\left( {16g_{1}^{4} n_{1}^{2} \left( {\gamma_{1}^{2} + \gamma_{1} \gamma_{2} + \gamma_{2} \left( {\gamma_{2} + 2\gamma_{3} } \right)} \right) + 4g_{1}^{2} n_{1} \gamma_{3} ((\gamma_{1} + \gamma_{2} )^{2} } \right. \\ & \quad \left. { + \left( {\gamma_{1}^{2} + 2\gamma_{2}^{2} } \right)} \right) + 4\left( { - \gamma_{2}^{2} + 3\gamma_{2} \gamma_{3} + \gamma_{3}^{2} + \gamma_{1} \left( {2\gamma_{2} + \gamma_{3} } \right)} \right)\left. {\Delta_{1}^{2} } \right) \\ & \quad + \gamma_{2} \left( {\gamma_{1} \gamma_{3}^{2} \left( {\gamma_{1}^{2} + 2\gamma_{2}^{2} } \right) + \gamma_{1} \left( {2\gamma_{2} + \gamma_{3} } \right)} \right) + 4\left( {\gamma_{1}^{3} + \gamma_{1}^{2} \gamma_{3} - 2\gamma_{2}^{2} \gamma_{3} } \right. \\ & \quad \left. { + \gamma_{3}^{3} + \gamma_{1} \gamma_{3} \left( { - 2\gamma_{2} + \gamma_{3} } \right)} \right)\left. { + 16\left( {\gamma_{1} + \gamma_{3} } \right)\left. {\Delta_{1}^{2} } \right)} \right), \\ \end{aligned}$$

26 $$ \begin{aligned} M_{4} & = 16\left( {n_{2} + 1} \right)^{2} \left( {4g_{1}^{2} n_{1} \left( {\gamma_{1}^{2} + \gamma_{2}^{2} + \gamma_{1} \left( {\gamma_{2} + \gamma_{3} } \right)} \right)} \right. \\ & \quad + \;\gamma_{2} \gamma_{3} \left( {2\gamma_{1}^{2} + \gamma_{2}^{2} + \gamma_{1} \left( {\gamma_{2} + 2\gamma_{3} } \right)} \right)\left. { - 8\gamma_{2} \left( {\gamma_{1} + \gamma_{3} } \right)\Delta_{1}^{2} } \right), \\ \end{aligned} $$

27 $$M_{6} = 64\left( {n_{2} + 1} \right)^{3} \gamma_{2} \left( {\gamma_{1} + \gamma_{3} } \right).$$

**Fig. 4:**
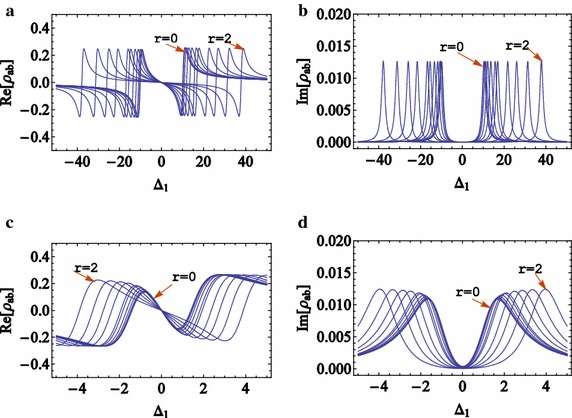
(Color online) **a** Dispersion and **b** absorption for different squeezing parameters and the number of coupling photons is n_2_ = 100. **c** Dispersion and **d** absorption for different squeezing parameters for vacuum coupling field (squeezing parameters are r = 0, 0.2, 0.4, 0.6, 0.8, 1, 1.2, 1.4, 1.6, 1.8, 2)

**Fig. 5:**
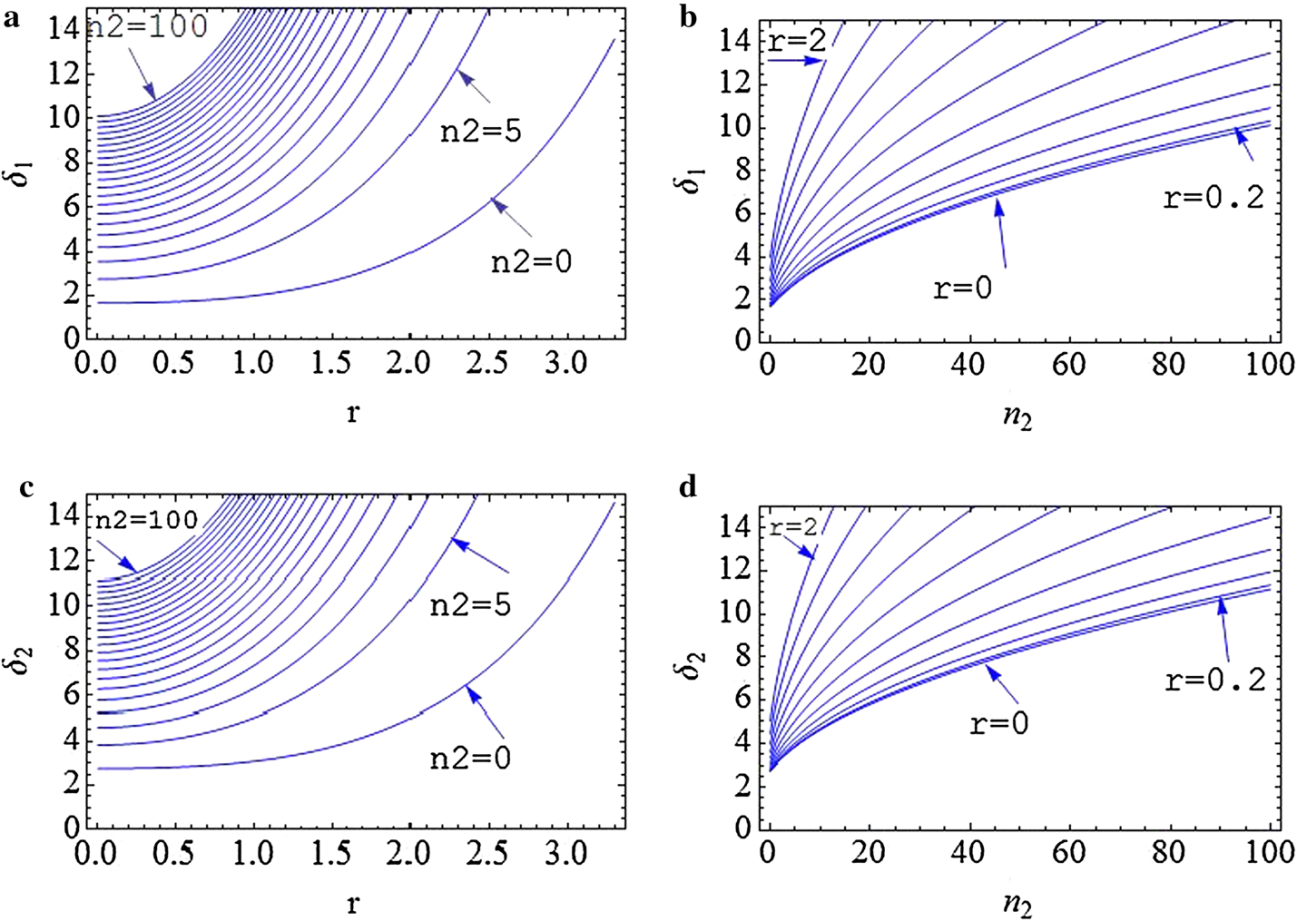
(Color online) **a** Variation of DAPs in terms of squeezing parameter for different number of coupling photons. **b** Variation of DAPs in terms of coupling photons for different values of squeezing parameters. **c** Variation of DDPs in terms of squeezing parameter for different number of coupling photons. **d** Variation of DDPs in terms of coupling photons for different values of squeezing parameters

The real and imaginary parts of the probe coherence term (20) correspond to the dispersion and absorption of probe photons, as plotted in Fig. 5a, b (Fig. 5c, d) for large (small) numbers of squeezed coupling photons for different squeezing parameters. The dispersion and absorption spectra drastically depend on the squeezing parameter and number of coupling photons, but are independent from the squeezing phase $\beta$. Figure 4a, c show that the DAPs and detuning of dispersion peaks (DDPs) are nonlinearly increased by increasing the squeezing parameter *r*, which leads to applying the photon counting method for a squeezing measurement by measuring the DAPs or DDPs.

By taking a derivative of the imaginary and real part of Eq. (20) and setting the results to zero, the DAPs and DDPs are obtained in terms of the number of coupling photons and their squeezing parameter. The DAPs and DDPs are plotted in terms of squeezing parameter for different numbers of coupling photons in Fig. 4a, c. DAPs and DDPs are more sensitive for larger squeezing parameter *r*. Furthermore, they are more sensitive to the squeezing parameter for a larger number of photons. Of course, it is important to note that *n*_2_ is not the average of coupling photons; but, it can be easily derived by $\bar{n}_{2} = \left\langle {n,\xi } \right|\hat{n}_{2} \left| {n,\xi } \right\rangle$. Figure 5b and d demonstrate the DAP and DDPs in terms of the number states for different squeezing parameters. It is similar to Fig. 3a which is useful for the photon counting and shows that the DAP and DDPs are more sensitive to the small number states where $r = 0$. This sensitivity is increased for a larger number states by increasing the squeezing parameter *r*. Therefore, number of coupling photons and their squeezing parameter can be obtained by measuring DAP and DDP of the absorption and dispersion spectra simultaneously. Some typical values of DAP and DDP are shown in Table 1 for different values of number of coupling photons and squeezing parameters. To measure the small number of photons or squeezing parameters, the accuracy of DAP and DDP measurements, according to the data in Table 1, should be about 0.2 g. For the range of atomic transition frequencies, 10 MHz < g < 1 THz the accuracy should be at least 2 MHz which is larger than the new electro-optical modulator resolutions (about 1 MHz) (Veisi et al. 2015).

**Table 1:** Different values of DAP ($\delta_{1}$) and DDP ($\delta_{2}$) in terms of some typical number of coupling photons (rows) and squeezing parameters (columns)

$$\begin{aligned} \delta_{1} , \hfill \\ \delta_{2} \hfill \\ \end{aligned}$$	0	1	2	3	4	5	50	100	150
0.0	1.666, 2.749	1.906, 2.993	2.140, 3.224	2.357, 3.437	2.557, 3.633	2.743, 3.817	7.244, 8.277	10.123, 11.148	12.348, 13.369
0.2	1.675, 2.758	1.926, 3.012	2.168, 3.251	2.390, 3.470	2.596, 3.672	2.787, 3.860	7.385, 8.419	10.323, 11.347	12.593, 13.614
0.4	1.704, 2.789	1.987, 3.073	2.525, 3.334	2.494, 3.571	2.715, 3.789	2.921, 3.991	7.815, 8.847	10.932, 11.956	13.340, 14.359
0.6	1.761, 2.847	2.097, 3.182	2.401, 3.481	2.674, 3.749	2.923, 3.993	3.153, 4.220	8.553, 9.582	11.975, 12.997	14.618, 15.636
0.8	1.854, 2.942	2.268, 3.349	2.627, 2.702	2.945, 4.015	3.233, 4.298	3.498, 4.559	9.628, 10.654	13.496, 14.515	16.479, 17.496
1.0	1.997, 3.083	2.511, 3.588	2.943, 4.013	3.321, 4.385	3.661, 4.721	3.973, 5.028	11.086, 12.110	15.555, 16.572	19.000, 20.015
2.0	3.956, 5.012	5.458, 6.501	6.629, 7.665	7.622, 8.654	8.450, 9.529	9.295, 10.322	26.895, 27.906	37.829, 38.837	46.247, 47.254

## Conclusions

In this paper, the master equations of Λ-type three-level atom interacting with two-mode quantized electromagnetic field and its exact coherence term are applied to obtain the squeezed and non-squeezed coupling photons. The following results were obtained: (1) The method was applied for presenting a photon counting and a squeezing measurement method by measuring the absorption spectrum of the probe photons. (2) The difference between the exact and WFA photon counting methods and benefits of the exact method (especially for the weak coupling photons) were demonstrated. This sensitivity increased for the larger number of coupling photons by increasing the squeezing parameter. (3) It was shown that the photon counting method was more sensitive for the smaller number of coupling photons. (4) The present method for the measurement of squeezing was more sensitive for larger values of squeezing parameters. (5) The number of coupling photons and their squeezing parameter can be obtained simultaneously by measuring the DAP and DDP. (6) The photon counting method was non-demolition for the strong coupling photons.

## Appendix

The Jaynes–Cummings Hamiltonian of a three-level Λ-type atom interacting with two mode quantized light in the interaction picture is given by Scully and Zubairy (1997):

28 $$\begin{aligned} V & = - \hbar g_{1} \left( {\sigma_{ab} e^{{i\omega_{ab} t}} + \sigma_{ba} e^{{ - i\omega_{ab} t}} } \right)\left( {a_{1} e^{{ - i\nu_{1} t}} + a_{1}^{ + } e^{{i\nu_{1} t}} } \right) \\ &\quad - \hbar g_{2} \left( {\sigma_{ac} e^{{i\omega_{ac} t}} + \sigma_{ca} e^{{ - i\omega_{ca} t}} } \right)\left( {a_{2} e^{{ - i\nu_{2} t}} + a_{2}^{ + } e^{{i\nu_{2} t}} } \right). \\ \end{aligned}$$

If one of the quantized mode of coupling photons be squeezed the interaction Hamiltonian will be transformed by a squeezing operator as:

29 $$\begin{aligned} V_{Squeezed} &= S_{ac}^{ + } VS_{ac}^{{}} = S_{ac}^{ + } ( - \hbar g_{1} (\sigma_{ab} e^{{i\omega_{ab} t}} + \sigma_{ba} e^{{ - i\omega_{ab} t}} )(a_{1}^{{}} e^{{ - i\nu_{1} t}} + a_{1}^{ + } e^{{i\nu_{1} t}} ))S_{ac}^{{}} \hfill \\& \quad + S_{ac}^{ + } ( - \hbar g_{2} (\sigma_{ac} e^{{i\omega_{ac} t}} + \sigma_{ca} e^{{ - i\omega_{ca} t}} )(a_{2}^{{}} e^{{ - i\nu_{2} t}} + a_{2}^{ + } e^{{i\nu_{2} t}} ))S_{ac}^{{}} , \hfill \\ \end{aligned}$$

where squeezing operator $\hat{S}(r) = \exp (\tfrac{1}{2}r\hat{a}_{2}^{2} - \tfrac{1}{2}r\hat{a}_{2}^{\dag 2} )$, and $r$ is the squeezing parameter. The squeezing operator acts on the second mode of creation and annihilation operators

30 $$\begin{aligned} V_{Squeezed} &= - \hbar g_{1} (\sigma_{ab} e^{{i\omega_{ab} t}} + \sigma_{ba} e^{{ - i\omega_{ab} t}} )(a_{1}^{{}} e^{{ - i\nu_{1} t}} + a_{1}^{ + } e^{{i\nu_{1} t}} ) \hfill \\ &\quad - \hbar g_{2} (\sigma_{ac} e^{{i\omega_{ac} t}} + \sigma_{ca} e^{{ - i\omega_{ca} t}} )(S_{ac}^{ + } a_{2}^{{}} S_{ac}^{{}} e^{{ - i\nu_{2} t}} + S_{ac}^{ + } a_{2}^{ + } S_{ac}^{{}} e^{{i\nu_{2} t}} ). \hfill \\ \end{aligned}$$

By applying the identities $S_{ac}^{ + } a_{2}^{{}} S_{ac}^{{}} = a_{2}^{{}} \text{Cosh} [r] - a_{2}^{ + } \text{Sinh} [r]$ and $S_{ac}^{ + } a_{2}^{ + } S_{ac}^{{}} = a_{2}^{ + } \text{Cosh} [r] - a_{2}^{{}} \text{Sinh} [r]$ and some straightforward calculations and rearrangement one obtains

31 $$\begin{aligned} V_{Squeezed} & = - \hbar g_{1} \left( {\sigma_{ab} a_{1} e^{{i(\omega_{ab} - \nu_{1} )t}} + \sigma_{ba} a_{1}^{{}} e^{{ - i(\omega_{ab} + \nu_{1} )t}} + \sigma_{ab} a_{1}^{ + } e^{{i(\omega_{ab} + \nu_{1} )t}} + \sigma_{ba} a_{1}^{ + } e^{{ - i(\omega_{ab} - \nu_{1} )t}} } \right) \\ & \quad -\, \hbar g_{2} \left[ {a_{2}^{{}} \sigma_{ac} \left( {\text{Cosh} [r]e^{{i(\omega_{ac} - \nu_{2} )t}} - \text{Sinh} [r]e^{{i(\omega_{ac} + \nu_{2} )t}} } \right)} \right. \\ & \quad +\, a_{2}^{ + } \sigma_{ac} \left( {\text{Cosh} [r]e^{{i(\omega_{ac} + \nu_{2} )t}} - \text{Sinh} [r]e^{{i(\omega_{ac} - \nu_{2} )t}} } \right) \\ & \quad +\, a_{2}^{{}} \sigma_{ca} \left( {\text{Cosh} [r]e^{{ - i(\omega_{ca} + \nu_{2} )t}} - \text{Sinh} [r]e^{{ - i(\omega_{ca} - \nu_{2} )t}} } \right) \\ & \quad \left. { +\, a_{2}^{ + } \sigma_{ca} \left( {\text{Cosh} [r]e^{{ - i(\omega_{ca} - \nu_{2} )t}} - \text{Sinh} [r]e^{{ - i(\omega_{ca} + \nu_{2} )t}} } \right)} \right]. \\ \end{aligned}$$

Because of the conservation of energy, the non-conservative terms, which contains $a\sigma^{ - }$, $a^{ + } \sigma^{ + }$ or $Exp\left[ {\omega + \nu } \right]$ should be removed to obtain Eq. (19), where $\Delta_{1} = \left( {\omega_{ab} - \nu_{1} } \right)$ and $\Delta_{2} = \left( {\omega_{ca} - \nu_{2} } \right)$.
